# Temperature-controlled radiofrequency energy in patients with anal incontinence: an interim analysis of worldwide data

**DOI:** 10.1093/gastro/gou016

**Published:** 2014-04-12

**Authors:** Richelle J.F. Felt-Bersma

**Affiliations:** Department of Gastroenterology and Hepatology, VU University Medical Centre, Amsterdam, Netherlands

**Keywords:** anal incontinence, radiofrequency, temperature control, SECCA

## Abstract

**Background**: Controlled delivery of radiofrequency energy (SECCA procedure) as treatment for anal incontinence (AI) was introduced 15 years ago. Since then, several clinical studies have emerged. This article evaluates the clinical response and sustainability of SECCA for patients with AI.

**Methods**: Only original clinical studies retrieved from PubMed and Medline were included. The outcome measures, faecal incontinence scores, definition of response, clinical results and anorectal evaluation were analysed.

**Results**: Ten studies were included, which involved 150 original patients. Three studies reported a long-term follow-up. The one-year follow-up shows a moderate effect, which declines somewhat over time. Only minor temporary side-effects are reported and none of the patients declined treatment.

**Conclusion**: SECCA is a safe and well-tolerated procedure that is easy to perform without any serious short- or long-term complications, but with only a moderate clinical effect that declines over time. Results of randomized, sham-controlled controlled trials are awaited.

## INTRODUCTION

Anal incontinence (AI) is defined as the loss or uncontrolled passage of liquid, solid stool or gas. It is a debilitating disorder causing diminished self-esteem, social isolation and stigmatisation. The prevalence of AI is estimated at 6–7% in the general population and rises with age—up to 20% in the elderly [1, 2]. Women are affected to a greater extent in earlier life and have a poorer comparative quality of life [3]. Although pathophysiological mechanisms of AI development often overlap, they can be categorized into five main causes: anal sphincter dysfunction, pudendal nerve neuropathy, poor rectal sensation, small rectal compliance and diarrhoea. In women, vaginal childbirth has been recognised as its main cause; this can be due to obstetric anal sphincter injury, stretch injury of the pudendal nerves or both. In males, incontinence is most frequently related to surgical procedures and proctitis after radiotherapy for prostate cancer [4, 5].

The initial management of AI starts with supplementation of dietary fibre, physiotherapy and the use of biofeedback techniques. These conservative treatments, especially when combined, are successful in the majority of patients [6, 7]. If unsuccessful, surgical procedures such as anal sphincter repair or sacral nerve stimulation (SNS) may be appropriate. The results of sphincter repair in patients with anal sphincter defects have been disappointing over the long term, leaving only 50% of patients with some response and acceptable continence status [8, 9]. SNS has been accepted as a treatment option for severe AI, with reasonable short- and long-term results. Recent 5-year follow-up studies have shown therapeutic success (defined as a >50% improvement of AI episodes per week) in 56–89% of patients undergoing implantation. Unfortunately, 20% of the referred patients are not suitable for an implant and, in 37% of patients, device revision, replacement or removal is required, underlining the invasive nature of the treatment [10–12]. Therefore new treatment options, such as the less-invasive SECCA procedure, are attractive. In this respect, SECCA delivers temperature-controlled radiofrequency (RF) energy in the anorectum for the purpose of improving the symptoms of AI. The supposed mechanism of SECCA is immediate collagen contraction, followed by wound healing and tissue remodelling, which results in a reduction of the rectal volume sensations, allowing the patient more time to reach the toilet. The first studies evaluating the procedure showed substantial reductions in incontinence scores; however, the true clinical benefit of these results still remains questionable [13]. This paper critically reviews the clinical efficacy, safety and anorectal function alterations that follow the SECCA procedure, both in the short and long terms, defining its role as a treatment option for severe AI.

## SECCA PROCEDURE

The SECCA procedure, which involves the administration of temperature-controlled RF energy to the anal canal, was first used for the treatment of AI in Mexico in 1999 by Curon Medical (Fremont, Ca, USA). The RF procedure, STRETTA, had previously shown a therapeutic effect in the treatment of gastro-oesophageal reflux. In 2002, the Food and Drug Administration (FDA) of the United States approved the SECCA system for use specifically in the treatment of patients with AI to solid or liquid stool, occurring at least once per week, and who already had failed to respond to more conservative therapies. According to the guidelines for the treatment of AI from the Practice Task Force of The American Society of Colon and Rectal Surgeons, the SECCA procedure is classified as a potentially useful intervention, based on Level III evidence due to the limited data regarding this treatment modality. In the European Union, more than 500 SECCA procedures have been performed since the re-launch of the technique in 2006 by Mederi Therapeutics (Greenwich, CT, USA). Currently, SECCA is performed in the United Kingdom, Germany, Italy, Spain and Turkey.

**Figure 1.**
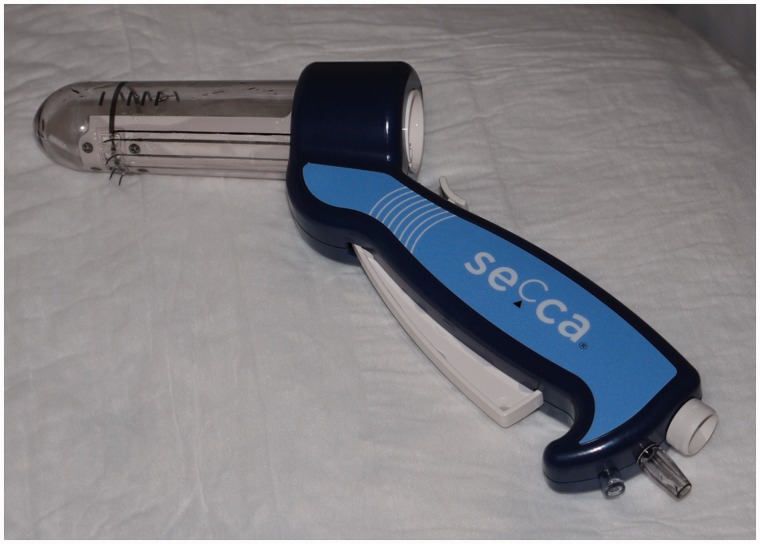
Secca® handpiece (probe).

**Figure 2.**
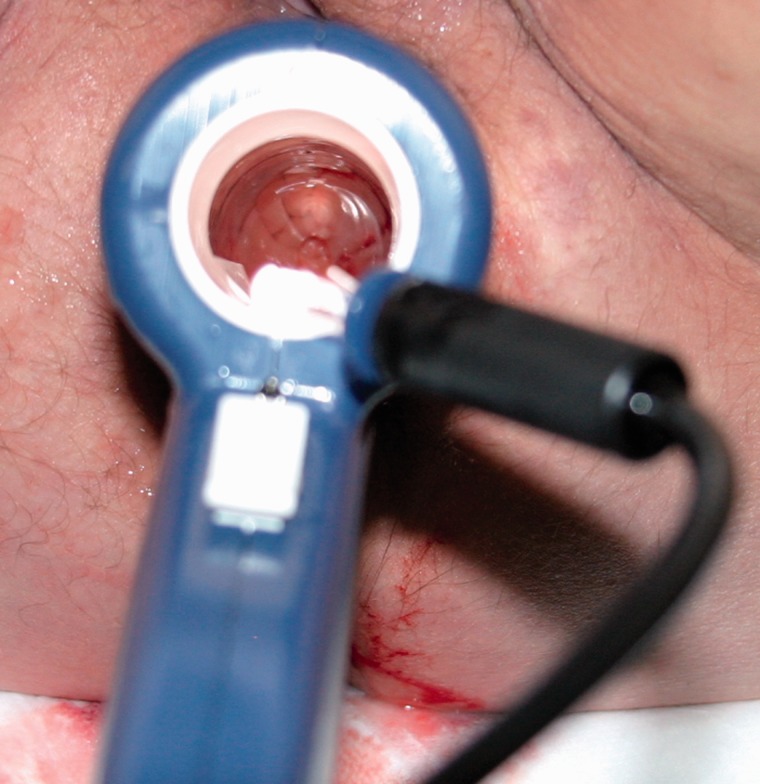
Probe application.

**Figure 3.**
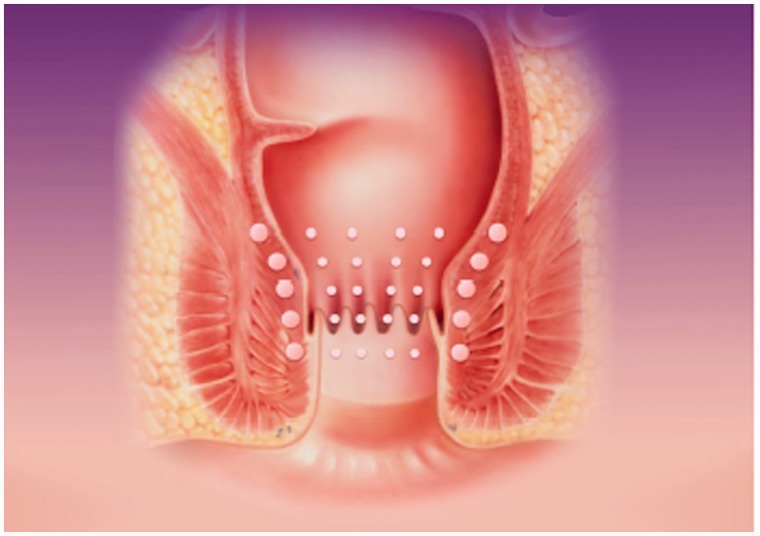
Schematic of the RF application points.

The SECCA procedure is generally performed under local anaesthesia and intravenous conscious sedation by means of fentanyl and midazolam. Antibiotics are generally administered and are aimed at Gram-rod and anaerobic bacteria. One or two hours prior to the procedure, patients are given a rectal enema. Patients are examined supine in the lithotomy or jack-knife position. The SECCA device is introduced into the anal canal, allowing good visual control of electrode placement. Once the applicator is satisfactorily in place, RF energy is delivered via four needles circumferentially in four quadrants at five different insertion levels each at 0.5 cm commencing at the dentate line upwards. The RF generator delivers energy at 465 kHz, 2–5 W, at each needle electrode for 90 seconds and power is automatically switched off when the temperature exceeds 85°C. The mucosa is constantly cooled by chilled water (45 mL/min) at the base of each needle. Each needle causes formation of five thermal lesions. This results in a total of 20 radiofrequency deliveries with 80–100 thermal lesions. The whole procedure takes about an hour. The technique is shown in Figures 1–3.

## SEARCH STRATEGY

A complete review of the available literature was conducted using the search engine targets “SECCA faecal incontinence” or “Radiofrequency faecal incontinence” assessing the PubMed and Medline databases. Abstracts and reviews were excluded. Most studies originated from a single treatment centre, whereas two were multi-centre studies [16, 20]. The articles were reviewed according to their outcome measures, faecal incontinence scores, definition of response, clinical results and anorectal evaluations. In this regard, anorectal functional evaluation and anal ultrasound were performed before and 3 months after the SECCA procedure in four studies [16, 17, 19, 22], two of which were conducted at the same centre [17, 22].

## OUTCOMES

There were 10 assessable clinical studies, as shown in Table 1, with a clinical responsiveness which varied widely from 6–84%, although the data are difficult to interpret as some studies objectively assessed improvements in patient scores (but collated the data), whereas others used more subjective determinations of response and visual analogue scales (VAS) [13–22]. Follow-up ranged from 3 months to 5 years. No differences in anorectal function or on ultrasound could be detected after treatment, with no specific parameters providing any discernible prognostic value. Complications were mild and varied from mucosal ulceration, delayed bleeding, local pain and diarrhoea (antibiotic-related) without any serious or life-threatening side-effects.

**Table 1.** Studies investigating the efficacy of the SECCA procedure

Literatures	*n*	Age in years (range)	Outcome measures	Follow- up	FIQL score improvement	Clinical response	Definition clinical response
Takahashi (2002)	10	56 (44–74)	CCF-AI, FIQL, SF-36	1 year	13.5–5	80%	>50% reduction in CCF-FI
Takahashi (2003)	10	56 (44–74)	CCF-FI FIQL, SF-36	2 years	13.8–7.3	70%	50% reduction in CCF-FI
Takahashi- Monroy (2008)	18	57 (44–74)	CCF-FI FIQL, SF-36	5 years	14.4–8.3	84%	>50% reduction in CCF-FI
Efron (2003)	50	61 (30–80)	CCF-FI FIQL, SF-36, VAS	6 months	14.5–11.1	60%	>10% improvement at VAS
Felt-Bersma (2007)	11	61 (49–73)	Vaizey score	1 year	18.8–15	55%	Subjective improvement
Lefebure (2008)	15	53 (33–72)	CCF-FI FIQL, SF-36, VAS	1 year	14.1–12.3	13%	>50% reduction in CCF-FI
Kim (2009)	8	61 (28–73)	FISI, FIQL	6 months	improvement	38%	Subjective improvement
Ruiz (2010)	24	73 (53–84)	CCF-FI, FIQL	1 year	15.6–12.9	12.5%	>50% reduction in CCF-FI
Abbas (2012)	27	44	CCF-FI	3 months 3.5 years	16–10.9 improvement	78% 22%	Subjective improvement
Visscher (2014)	24	59 (44–73)	Vaizey score, FIQL	3 years	18–14	6%	≥50% reduction of Vaizey score

*n* = number of patients; CCF-FI = Cleveland Clinic Florida Faecal Incontinence Scale; FIQL = Faecal Incontinence-related Quality of Life Score; SF-36 = Short Form-36 Scale; VAS = Visual Analogue Scale; FISI = Faecal Incontinence Severity Index

The initial study of SECCA use showed a remarkably good response, which was never repeated in later series [13]. After one year, improvement effectively ranged from 12.5–80%, with the criteria used being generally >50% of the Cleveland Clinic Florida Faecal Incontinence Scale (CCF-FI) [10, 13–16, 18, 22], or a subjective improvement by means of a VAS scale [17, 19, 21]. Only three studies showed a long-term reported follow-up [15, 21, 22]. In this respect, the 5-year follow-up of the initial study [13] showed an exceptional sustained response, while the two other longer-term follow-up studies showed a preserved faecal continence ranging from 6–22% after more than three years of assessment [21, 22]. In those studies reporting on quality of life (QoL) the scores of the faecal incontinence QoL subscales—including lifestyle, coping, depression and embarrassment—all improved significantly from 0.5–1 point on a 4-point scale in each parameter in some studies, [13, 16] in three of the domains in one study [20] and in only one of the QoL domains in another study [18], with the SF-36 QoL scale generally showing improvement. The overall results of the AI scores paralleled those of the reported QoL scores.

Individual analysis of the available data on this procedure remains limited, with Kim *et al.* from Korea who showed no clinical benefit in the faecal incontinence severity index or the QoL parameters in eight patients [19], with a moderate reported incidence of pain and bleeding after the procedure. Fifteen procedures performed by Lefebure *et al.* from Rouen, France, showed minimal, non-significant improvement in the Wexner scores after SECCA and a slight improvement in the depression scale of QoL assessment [18]. Takahashi's later results, published in 2008 from Mexico, on 19 patients treated over 5 years to show durability of response in longer-term follow-up, resulted in a small but significant improvement in all QoL and AI parameters [15]. In a study by our group, 6 of 11 patients improved clinically at 3 months, the effect persisting at one year [17]. Anorectal function tests showed that there was no difference in anorectal manometry or simple compliance assessment in this study, although there were slight improvements in rectal distension volumes to urge and in maximal tolerance to balloon distension. A 5-centre study enrolling 50 patients, reported by Efron from the Cleveland Clinic Florida, showed an improvement in the CCF-FI scores and in all the QoL coping parameters at 6 months [16]. Data from Ruiz *et al.*, concerning 24 patients with a 12-month follow-up, showed slight improvement in the CCF-FI scoring and in the depression scale for treated cases [20], whilst our own group's latest data on 24 patients showed poor results over a medium-term follow-up in objective definitions of success on an intention-to-treat basis [22].

## DISCUSSION

RF energy delivered to the anal canal is a surgical modality used to treat AI and is an extension of its use in other conditions, including prostatic hypertrophy, gastro-oesophageal reflux disease, sleep apnoea syndrome and the ablation of hepatic tumours. SECCA clearly shows a great variability of response using different response criteria. There are several reasons for this response disparity. Firstly, the definition of improvement plays a role. Subjective improvement or 10% improvement is different from a 50% reduction in the CCF-FI score. Further, in the initial study, not all patients went through a regimen of maximum conservative treatment of fibre and physiotherapy or biofeedback, therefore less-severe patients were included for analysis. Other studies included only patients who failed on conservative measures, therefore selecting more-severe patients. In spite of the significant improvement, it is also contentious as to what an increase from 20 to 10 on the CCF-FI scale means to patients, since they remain substantially incontinent.

The mechanisms by which SECCA could improve continence are unclear. Its principle is similar to the controlled delivery of RF energy to the lower oesophageal sphincter region (the STRETTA procedure), which was proposed as an alternative to standard anti-reflux surgery in patients with gastro-oesophageal reflux disease. It has been suggested that the beneficial effect from SECCA may be due to a tightening of the anorectum after a controlled fibrosis, which results in a reduction of rectal volume sensations [17]. As a consequence, the patient senses distension earlier and therefore has additional time to reach the toilet. However, no study has been able to show a significant difference in rectal sensation induced by SECCA treatment. Furthermore, there is no explanation why some patients who do respond to SECCA seem to lose their improvement over time, since it is likely that the reactive fibrosis would have stabilized the rectal sensation. It is most likely that the ongoing pathological process causing the incontinence or an advancing pudendal neuropathy and loss of supporting tissues sufficiently worsens, resulting in clinical deterioration independent of SECCA treatment.

In summary, SECCA is can be applicated in selected cases, with an excellent safety profile and very few side-effects. Even though clinical response is achieved in a minority, the minimally invasive aspect of the technique and its safety, as well as its low cost and its positive effect on incontinence-related quality of life parameters, may suggest its use as a temporary option for selected patients with moderately severe AI. Unfortunately, at the present time, there are no predictive factors regarding success or failure of treatment. The author would suggest that there is evidence of a clinical response of a seemingly temporary nature in a minority of patients so treated, but also a need for randomised, sham-controlled trials (which are ongoing) designed to differentiate between a true beneficial effect and a placebo response.

## FUNDING

**Conflict of interest**: none declared. The SECCA devices are manufactured by Mederi Therapeutics. However, Mederi Therapeutics did not play any role in the design and conduct of the study. Furthermore, Mederi Therapeutics neither has any input into the review of the results and writing this manuscript.
